# Stereotactic radiation therapy for oligometastases or oligorecurrence within mediastinal lymph nodes

**DOI:** 10.18632/oncotarget.7636

**Published:** 2016-02-23

**Authors:** Huan-Huan Wang, Nicholas G. Zaorsky, Mao-Bin Meng, Xian-Liang Zeng, Lei Deng, Yong-Chun Song, Hong-Qing Zhuang, Feng-Tong Li, Lu-Jun Zhao, Zhi-Yong Yuan, Ping Wang, Xi-Shan Hao

**Affiliations:** ^1^ Department of Radiation Oncology, CyberKnife Center, and Key Laboratory of Cancer Prevention and Therapy, Tianjin Medical University Cancer Institute & Hospital, National Clinical Research Center for Cancer, Tianjin, China; ^2^ Department of Radiation Oncology, Fox Chase Cancer Center, Philadelphia, PA, USA; ^3^ Department of Thoracic Cancer and Huaxi Student Society of Oncology Research, West China Hospital, West China School of Medicine, Sichuan University, Chengdu, China; ^4^ Department of Gastrointestinal Surgery, Key Laboratory of Cancer Prevention and Therapy, National Clinical Research Center for Cancer, Tianjin Medical University Cancer Institute & Hospital, Tianjin, China

**Keywords:** stereotactic radiation therapy, mediastinal lymph node, neoplasm metastasis, oligometastasis, oligorecurrence

## Abstract

**Aims:**

This study evaluated the safety and efficacy of stereotactic radiation therapy (SRT) for the treatment of patients with oligometastases or oligorecurrence within mediastinal lymph nodes (MLNs) originating from different tumors.

**Methods:**

Between October 2006 and May 2015, patients with MLN oligometastases or oligorecurrence were enrolled and treated with SRT at our hospital. The primary endpoint was MLN local control (LC). Secondary endpoints were time to symptom alleviation, overall survival (OS) after SRT, and toxicity using the Common Terminology Criteria for Adverse Events (CTCAE v4.0).

**Results:**

Eighty-five patients with 98 MLN oligometastases or oligorecurrences were treated with SRT. For the entire cohort, the 1-year and 5-year actuarial LC rates were 97% and 77%, respectively. Of 53 symptomatic patients, symptom alleviation was observed in 47 (89%) after a median of 5 days (range, 3-30 days). The median OS was 27.2 months for all patients. For patients with non-small cell lung cancer, univariate and multivariate analyses revealed that a shorter interval between diagnosis of primary tumors and SRT and larger MLN SRT volume were associated with worse OS. CTCAE v4.0 ≥ Grade 3 toxicities occurred in six patients (7%), with Grade 5 in three patients (all with RT history to MLN station 7).

**Conclusions:**

SRT is a safe and efficacious treatment modality for patients with oligometastases or oligorecurrence to MLNs originating from different tumors, except for patients who received radiotherapy to MLN station 7. Further investigation is warranted to identify the patients who benefit most from this treatment modality.

## INTRODUCTION

Oligometastasis and oligorecurrence refer to a state in which a patient has a limited number of distant metastatic regions (typically ≤5) that contain the primary tumor. These states may be noted at the time of diagnosis (i.e. oligometastsis) or as failure after definitive therapy (i.e. oligorecurrence). Although most patients with distant metastases are typically incurable, the oligometastatic/oligorecurrent state implies that metastasis-directed therapy may cure the disease (e.g. liver metastasis from colorectal cancer) [1].

As of 2016, there is no standard approach for managing patients with oligometastases or oligorecurrence. Conventional therapy for those with oligometastatic/recurrent disease is systemic therapy alone (e.g. with chemotherapy, hormones, or targeted agents); unfortunately, this rarely eradicates gross disease. Moreover, surgical salvage of these patients is not always feasible, given their proclivity to juxtapose critical structures (e.g. esophagus, great vessels, and trachea) [2].

Over the last decade, evidence has emerged suggesting patients with oligometastases or oligorecurrence may be cured with metastasis-directed stereotactic radiation therapy (SRT) [3-10]. SRT is a type of external beam radiation therapy (EBRT) that delivers RT accurately and precisely to the tumor, with fewer fractions and higher biologically equivalent dose (BED) than conventionally fractionated radiation therapy. SRT is divided into stereotactic body RT (SBRT; the delivery of 3.5-15 Gy per fraction, in 5 fractions or less) and fractionated stereotactic RT (FSRT; with delivery in more than 5 fractions). SRT can be delivered using either a traditional linear accelerator or a robotic arm (i.e. CyberKnife).

Our preliminary report on SRT for recurrent/secondary primary mediastinal lymph node (MLN) metastases from non-small cell lung cancer (NSCLC) revealed that SRT appears to be a safe and efficacious treatment modality for patients without previous RT [11]. The purpose of the current study is to update our previous report and evaluate the safety and efficacy of SRT for patients with oligometastases or oligorecurrence to MLNs originating from different tumors.

## RESULTS

### Patient and treatment characteristics

Clinical information on 3,332 patients with different primary or metastatic tumors treated with SRT between October 1, 2006 and May 1, 2015 at the CyberKnife Center of Tianjin Medical University Cancer Institute & Hospital was reviewed. Of all patients, there were85 patients with 98 MLN oligometastases or oligorecurrences treated with SRT. The median interval from the diagnosis of primary cancer to the first day of SRT was 21.2 months (range, 0.5 - 305.7 months). Among the patients, 72 had one MLN oligometastasis or oligorecurrence, and thirteen patients had two MLN oligometastases or oligorecurrences within one MLN zone. Thirty-seven patients (37/85, 44%) had synchronous oligometastases or oligorecurrence, and localized treatment was applied to all lesions.

**Figure 1:**
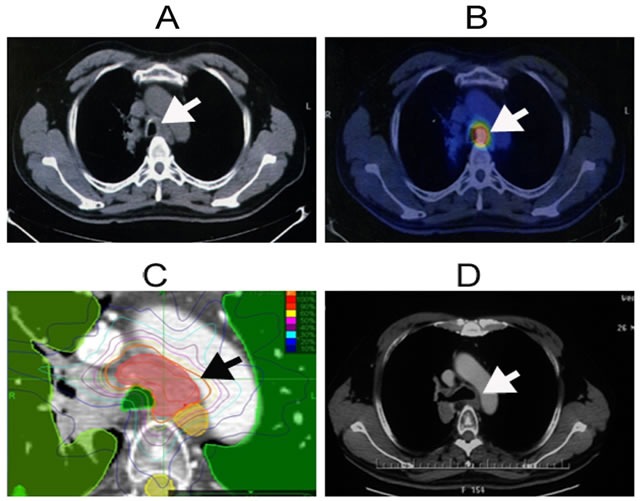
An illustrative case of successful SRT for oligometastatic NSCLC to a MLN 52-year-old man with squamous cell carcinoma in the upper lobe of the left lung. Images were taken after surgery and systematic lymphadenectomy; he was treated for a station 4 MLN oligorecurrence with SRT prescribed at 48 Gy in 8 fractions. The pretreatment CT **A.** and PET-CT **B.** showed the hyperactive metabolic activity in the station 4 MLN oligorecurrence. **C.** The planning CT and isodose distributions with SRT and contours depict planning target volume (red), lung (green), esophagus and cord (yellow), and bronchus (green). **D.** Post-SRT CT showed complete response. MLN: mediastinal lymph node; SRT: stereotactic radiation therapy; CT: computed tomography; PET-CT: positron emission tomography- computed tomography. Note: The arrows indicate MLN oligorecurrence.

**Figure 2:**
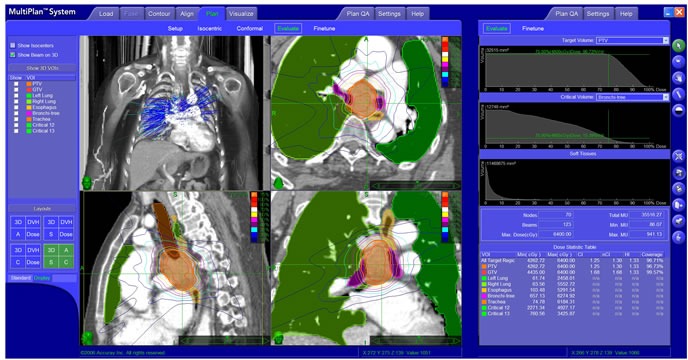
An illustrative case of Grade 5 toxicity after SRT for oligometastatic NSCLC to a station 7 MLN This patient, a 64-year-old woman with squamous cell lung cancer located in her left lower lobe with station 7 MLN, received SRT 6.8 months after completion of definitive RT. SRT was 48 Gy in 8 fractions, prescribed to the 75% isodose line, which covered 95% of the PTV. The PTV was also amended to avoid adjacent organs at risk (i.e. esophagus, brachial, trachea, spine cord, and heart). The outermost line is the 30% isodose line (outermost blue line). Unfortunately, the patient died from a tracheoesophageal fistula six weeks after completion of SRT, despite meeting dose constraints. MLN: mediastinal lymph node; RT: radiation therapy; SRT: stereotactic radiation therapy; Gy: Gray; GTV: gross tumor volume; CT: computed tomography; PTV: planning target volume.

**Figure 3:**
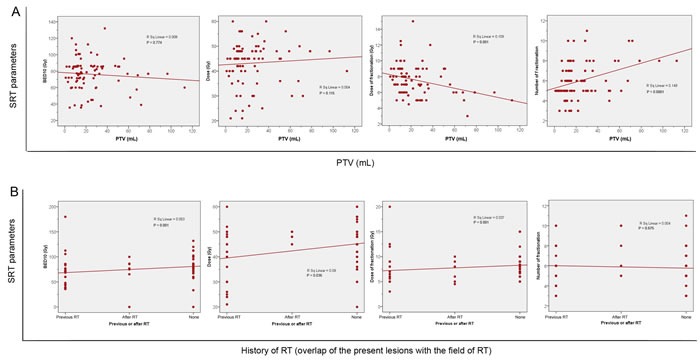
Scatterplots of: **A.** The relationship between SRT parameters and MLN PTV. **B.** The relationship between SRT parameters and patients with history of RT. Some patients first received SRT and then received conventionally fractionated RT; others first received conventionally fractionated RT and then received SRT; others received SRT alone. SRT: stereotactic radiation therapy; RT: radiation therapy; BED: biologically equivalent dose.

The patient and treatment characteristics of all patients and NSCLC patients are shown in Table 1 and Supplementary file 2, respectively. The radiation doses to normal organs (e.g. trachea, esophagus, aorta) are shown in Supplementary file 3. An example of successful treatment plan is shown in Figure 1. A treatment plan where the patient experienced Grade 5 toxicity (despite meeting dose constraints) is shown in Figure 2. Table 2 provides a detailed summary of the MLN stations and SRT treatment planning parameters for all patients. Figure 3 shows the relationships of various SRT parameters.

**Table 1:** Summary of patient and treatment characteristics (*N*=85)

Parameter	No. (%)
Age (years) at the SRT	
Median (range)	59 (32-89)
Gender	
Male	54 (64)
Female	31 (36)
Primary tumors	
Non-small cell lung cancer	53 (62)
Esophageal carcinoma	7 (8)
Breast cancer	7 (8)
Hepatocellular carcinoma	3 (4)
Thyroid carcinoma	3 (4)
Kidney cancer	3 (4)
Bladder cancer	2 (2)
Thymic carcinoma	1 (1)
Nasopharyngeal cancer	1 (1)
Ovarian cancer	1 (1)
Rectal cancer	1 (1)
Cervical cancer	1 (1)
Sublingual adenocarcinoma	1 (1)
Submandibular gland cancer	1 (1)
No. of MLNs within LN zone	
1	72(85)
2	13 (15)
Clinical symptoms of MLNs	
Yes	53 (62)
No	32 (38)
Radiographic diagnosis of MLNs	
PET-CT	64 (75)
CT	21 (25)
Synchronous metastases	
Yes	37 (44)
No	48 (25)
Interval between the diagnosis of primary and SRT (median, in months)	
< 21.23	49 (58)
≥ 21.23	36 (42)
History ofRT (overlap of the present lesions with the field of RT)	
Yes	29 (34)
No	56 (66)
SRT treatment intent	
Curative	83 (98)
Palliative	2 (3)
Other treatments after SRT	
Chemotherapy	37 (48)
Endocrine therapy^†^	3 (4)
Molecular targeted therapy^‡^	9 (11)

† Three breast cancer patients with positive of ER, PR, and Her-2 received endocrine therapy.

‡ Seven lung adenocarcinoma patients with EGFR mutation received erlotinib or gefitinib, and 2 hepatocellular carcinoma received sorafenib.

Abbreviations: MLNs: mediastinal lymph nodes; PET-CT: positron emission tomography/computed tomography; CT: computed tomography; SRT: stereotactic radiation therapy; RT: radiotherapy.

**Table 2:** MLN stations and SRT treatment parameters

Nodal zone	No. (%)†	PTV (mL)	Prescription dose (Gy)	No. of fractions	Dose per fraction (Gy)	BED10 (Gy)	Prescription isodose line (%)
Upper	46 (47%)						
1R	5 (11%)	25.9 (13.5-28.3)	45 (42-48)	6 (3-7)	7 (6.86-15)	80.9 (71.4-112.5)	70 (65-75)
1L	4 (9%)	27.0 (3.0-34.0)	36.5 (25-48)	5 (5-6)	7.3 (5-8)	73.0 (37.5-86.4)	78.5 (65-81)
2R	14 (30%)	16.0 (0.9-68.7)	45 (21-50)	5.5 (3-10)	6.93 (5-10)	72 (35.7-100)	73.5 (62-81)
2L	3 (7%)	31.3 (26.6-36.0)	45 (45-52)	5 (5-8)	9 (6.5-9)	85.5 (85.5-85.8)	67.5 (63-72)
3A	6 (13%)	34.3 (13.8-51.3)	45 (30-60)	5 (3-5)	9 (8-12)	85.5 (60-132)	79.5 (76-82)
4R	8 (17%)	11.5 (4.4-50.6)	45 (36-50)	5 (3-6)	9 (7-15)	85.5 (68-112.5)	76 (70-81)
4L	6 (13%)	14.9 (5.9-18.1)	45 (35-49)	5.5 (5-8)	7.5 (6-8.4)	77.0 (59.5-86.4)	71.5 (66-74)
Aorticopulmonary	21 (21%)						
5	17 (81%)	13.3 (1.7-112.8)	42 (24-50)	5 (3-10)	8.4 (4.5-10)	76.8 (38.4-100)	74 (67-81)
6	4 (19%)	18.6 (15.3-34.4)	45 (21-45)	4 (3-6)	8.25 (7-15)	82.1 (35.7-112.5)	73.5 (63-79)
Subcarinal	9 (9%)						
7	9 (100%)	12.6 (7.7-71.8)	45 (24-50)	7 (4-10)	6 (3-9)	76.8 (38.4-85.5)	74 (66-82)
Lower	7 (7%)						
8	2 (29%)	19.4 (10.5-28.3)	50 (50, 50)	7 (4, 10)	8.75 (5, 12.5)	93.75 (75, 112.5)	79.5 (79, 80)
9	5 (71%)	18.4 (6.8-78.5	45 (30-60)	6 (3-8)	6 (5-20)	76.8 (45-180)	77.5 (77-80)
Hilar-interlobar	15 (15%)						
10R	8 (53%)	26.3 (10.6-67.6)	52 (36-56)	6 (4-8)	8 (6-12)	100.4 (57.6-105.6)	75 (65-78)
10L	7 (47%)	35.2 (6.6-96.6)	49 (30-60)	7 (5-11)	7 (5-10)	83.3 (48-120)	76 (70-82)
All	98 (100%)						

† Number of MLN metastases.

Abbreviations: MLNs: mediastinal lymph nodes; R: right; L: left. SRT: stereotactic radiation therapy; PTV: planning target volume; Gy: Gray; BED_10_: biologically equivalent dose at α/β value of 10.

Note: all numbers presented are medians (with range or percent in parenthesis).

### MLN response and time to symptoms alleviation

**Figure 4:**
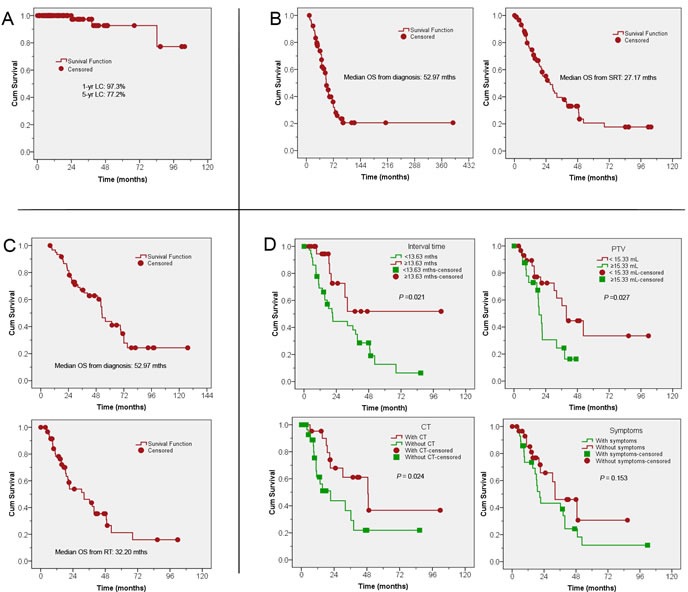
Actuarial LC and OS from time of diagnosis and from SRT for patients with oligometastases or oligorecurrence to MLNs **A.** Actuarial LC for all patients; **B.** OS from diagnosis and from SRT for all patients; **C.** OS from diagnosis and from SRT for patients with NSCLC; **D.** OS for NSCLC patients by: interval time between diagnosis of the primary tumor and SRT (upper left); MLN PTV (upper right); presence or absence of CT (lower right); and presence or absence of symptoms (lower left). LC: local control; OS: overall survival; SRT: stereotactic radiation therapy; MLN: mediastinal lymph node; NSCLC: non-small cell lung cancer; PTV: planning target volume; CT: chemotherapy.

Clinical tumor responses were evaluated at 6 months after SRT using CT and/or PET-CT scans. Out of the 85 patients, 74 (74/85, 87%) had a CR, five (5/85, 6%) had PR, three (3/85, 4%) had SD, and three (3/85, 4%) had PD. The 1-year and 5-year actuarial LC rates for all eligible patients were 97% and 77%, respectively (Figure 4A). Symptom alleviation was observed in 47 out of 53 patients (89%) after a median of 5 days (range, 3-30 days), lasting through the follow-up period. Of these 47 patients, there was complete resolution of symptoms and discontinuation of medications in 42 patients.

### Overall survival

For the whole cohort, median follow-up was 42.2 months (range, 8.1-389.9 months). The median OS from diagnosis and from SRT, respectively, was: 53.0 months and 27.2 months for all patients (Figure 4B); 52.3 months and 32.2 months for NSCLC (Figure 4C).

The following analyses were carried out for the 53 NSCLC patients only, since they made up the majority of the primary tumors. Further characteristics of these patients are listed in Supplementary File 2. The 1-, 3-, and 5-year OS rates from SRT were 78.2%, 43.6%, and 21.3%, respectively. A worse OS was associated with a shorter time until SRT, larger PTV size, or non-use of chemotherapy (Figure 4D). Differences in OS from SRT were insignificant for presence vs. absence of symptoms (*p* = 0.15), synchronicity of oligometastases or oligorecurrence (*p* = 0.26), number of lesions (*p* = 0.25), MLN location of lesions (*p* = 0.48), SRT treatment intent (*p* = 0.73), and patient history of RT (*p* = 0.12). On multivariate analysis (Table 3) for NSCLC patients, a worse OS was noted for patients with shorter interval time between diagnosis of primary tumors and SRT (median, 13.6 months) and a larger MLN PTV (median, 15.3 mL).

**Table 3:** Multivariate subgroup analyses for OS from SRT in NSCLC

Parameters	HR	95% CI	*p* values
Interval time (< 13.63 months vs. ≥ 13.63 months)	0.256	0.083-0.791	0.018
PTV volume (< 15.33 mL vs. ≥ 15.33 mL)	3.656	1.348-9.913	0.011
CT after SRT (yes vs. no)	1.549	0.559-4.008	0.367

Abbreviations: OS: overall survival; NSCLC: non-small cell lung cancer; HR: hazard ratio; CI: confidence interval; SRT: stereotactic radiation therapy; PTV: planning target volume; CT: chemotherapy.

### Patterns of failure

Among all eligible patients three (3/85, 4%) relapsed within the PTV. These patients had disease in MLNs of 2R, 10R, or 1L. Forty-nine patients (49/85, 58%) had out-of-field progression with a median of 9.6 months after SRT (range, 0.5-85.6 months); 33 patients (33/85, 39%) had no progression after SRT. Among the patients with progression, two (2/85, 2%) had diffuse progression including regional failure. These two patients had distant metastases to lung, bone, brain, liver and non-regional lymph nodes.

### Toxicities

Toxicity of all patients and the radiation dose for trachea, esophagus, heart, aorta, and brachial plexus are summarized in Table 4 and Supplementary file 3, respectively. Eleven patients (11/85, 13%) experienced CTCAE v4.0 Grade 1 to 2 acute toxicities. Four patients (4/85, 5%) experienced Grade 3 pneumonitis, esophagitis, and/or tracheitis; these Grade 3 toxicities were generally transient and resolved with conservative management. Late radiation toxicities were observed in six patients (6/85, 7%); three of them (3/85, 4%) died from Grade 5 late radiation toxicities (either tracheoesophageal or esophageal-mediastinal fistula), all of which had history of RT to MLNs in station 7.

**Table 4:** Toxicities of patients with oligometastases or oligorecurrence to MLNs treated with SRT

	Total, n (%)			
Acute toxicities	Any Grade	Grade 3	Grade 4	Grade 5
Pneumonitis	6 (7)	1 (1)	0	0
Esophagitis	3 (4)	1 (1)	0	0
Tracheitis	3 (4)	2 (2)	0	0
Chest pain	1 (1)	0	0	0
Agranulocytosis	1 (1)	0	0	0
Thrombocytopenia	1 (1)	0	0	0
Late toxicities				
Tachycardia	1 (1)	0	0	0
Lung fibrosis	1 (1)	0	0	0
Atelectasis	1 (1)	0	0	0
Tracheoesophageal fistula	2 (1)	0	0	2 (2)
Esophageal-mediastinal fistula	1 (1)	0	0	1 (1)

Abbreviations: MLNs: mediastinal lymph nodes; SRT: stereotacticradiation therapy.

## DISCUSSION

To our knowledge, this is the largest study to evaluate the safety and efficacy of SRT for patients with oligometastases or oligorecurrences of various tumors to MLNs. Our results demonstrate that SRT is a safe and efficacious treatment modality for such patients, except for those who had history of RT to MLN station 7. Further investigation is warranted to identify the patients who benefit most from this treatment modality.

MLN oligometastasis and oligorecurrence are common in NSCLC, occurring in 20% of patients with stage I disease and up to 50% of patients with stage III disease. The majority of failures are confined to the thorax, and survival after recurrence is < 30% [12-13]. SRT appears to be a promising treatment option for most patients [14-18] (Figures 1 and 2). In this study, the majority of patients (53/85, 62%) had MLN oligometastases or oligorecurrence from NSCLC (Table 1), and we performed a subset analyses of these patients. Notably, more than 30% of patients had oligorecurrence in MLNs typically inaccessible by minimally invasive techniques (e.g. levels 2-7), but allowing achievement of high BED_10_s, typically > 100 Gy (Table 2).

Our results are consistent with previous studies which suggest that worse OS is associated with short interval since previous RT, poor patient performance status, large target volume, and previous RT to the adjacent critical structures (Figure 3) [11, 19-29]. However, we did not note worse LC with larger PTVs, as seen in other studies [20, 28], perhaps because we were generally able to maintain BED_10_s >100 (Table 2). The 1-year and 5-year actuarial LC rates for all eligible patients (97% and 77%, respectively) were higher than reported rates for conventional RT (typically < 65% at 2 years) [30].

The median OS for our NSCLC patients (32 months) is superior to the OS of those treated with conventional RT (11 - 19 months) [31] and of unresectable IIIA and IIIB NSCLC patients receiving concurrent chemo-RT (16 - 19 months) [32-34]. We hypothesize that the longer OS is due to a longer cell replication time (as suggested by longer time from diagnosis to SRT), small disease burden (as suggested by smaller PTV) and improved patient performance status (as suggested by patients ability to receive chemotherapy after SRT) (Table 3). Thus, we believe that oligometastatic/oligorecurrent patients are a distinct subset of metastatic patients because many may be cured of their disease. Investigating novel curative local therapies that have minimal toxicity is important for these patients.

The low-toxicity profile (Table 4) observed in our study is of particular importance in cancer patients, who have received or will receive other oncologic therapies. The majority of our patients (> 90%) experienced CTCAE Grade 1-2 acute or late toxic events; most of these symptoms were transient and resolved with conservative management. However, despite meeting normal tissue constraints, three patients (4%) died from Grade 5 late toxicities, all of which had history of RT to LN station 7. Our results corroborate those of Corradetti *et al* [35] nd we advise extreme caution when considering SRT (especially re-irradiation) around the mainstem bronchi (e.g. level 7 LNs), as this may cause central airway necrosis or tracheoesophageal fistula, as seen in the current study.

This study has limitations, including its retrospective nature, inclusion of a heterogeneous group of patients with different primary tumors, different MLN treatment sites, curative vs. palliative treatment intent, disease extent, RT history, fractionation regimens, and systemic therapies used. We can only draw associations but not causation between patient outcomes and treatment characteristics.

Further studies are needed to evaluate confounders, including age, comorbidity, performance status, histology, the primary tumor site, and genetic differences between the primary tumor and metastases. For example, certain patients may have a better performance status and may tolerate more systemic therapies (as seen in Figure 3). Thus, we recommend clinicians treat patients based on a personalized, multidisciplinary approach for each patient.

In conclusion, SRT is a safe and efficacious for patients with oligometastases or oligorecurrences to MLNs originating from different tumors, though we recommend caution in re-irradiation to MLN station 7. Palliation of symptoms is achievable in most patients with symptomatic lesions. Further investigation is warranted to identify the patients who benefit most from this treatment modality.

## PATIENTS AND METHODS

### Study design and eligible patients

We retrospectively queried our prospectively-collected database of patients with oligometastases or oligorecurrence to a MLN, treated between October 1, 2006 and May 1, 2015. All patients were examined in a multidisciplinary setting at the time of diagnosis or recurrence, and their cases were re-presented to the tumor board as needed. The inclusion criteria were: (i) any age; (ii) Karnofsky performance score (KPS) ≥ 70; (iii) oligometastasis or oligorecurrence to one or two MLNs within the MLN zone; (iv) any primary tumor site, with prior biopsy and histologic confirmation and either computed tomography (CT) or positron emission tomography (PET)-CT images; (v) life expectancy ≥ six months; (vi) unamenable to resection (either because of anatomical tumor characteristics or patient comorbidities); and (vii) patient written informed consent for the treatment and inclusion in the database. Exclusion criteria were contraindication for receiving RT and uncontrolled comorbid condition (metabolic or psychiatric). The study protocol was in accordance with the ethical guidelines of the 1995 Declaration of Helsinki and was approved by the independent ethics committees at Tianjin Medical University Cancer Institute & Hospital, National Clinical Research Center for Cancer, China.

### Treatment

MLN stations were classified according to Mountain and Dresler and were delineated following the atlas from the University of Michigan [36-37]. The methodology used for CyberKnife SRT and treatment planning was as described in our preliminary study [11]. Briefly, patients were immobilized using a vacuum bag before CT simulation. A set of planning three- and four-dimensional (3D/4D) CT images were obtained with IV contrast to highlight the MLN metastases. The gross target volume (GTV) was defined for the MLN disease based on simulation, CT, and/or PET-CT. The planning target volume (PTV) was defined as the GTV with a margin of 0.3 cm. The PTV was also amended to avoid adjacent organs at risk (i.e. esophagus, brachial, trachea, spine cord, heart). The Xsight spine tracking system was used for positional alignment based on bony spinal skeletal structures.

BEDs were calculated based on the formula: nd[1 + d/(α/β)], where n is number of fractions, and d is dose/fraction (Gy); assuming α/β value of 10 for lung cancer or acute toxicities (i.e. BED_10_), and assuming α/β value of 3.0 for late toxicities (i.e. BED_3_). Our treating physicians typically try to increase dose of PTV BED_10_ to > 100 Gy, as this has been shown to be associated with improved LC [38-41]. Unfortunately, delivery of a high BED to the tumor is not always possible, given the juxtaposition of PTV to critical structures (e.g. great vessels, esophagus). Moreover, for all patients with previous RT, the original treatment plans are incorporated and all BEDs were summed (in order to minimize the dose to critical organs), limiting the prescribed doses further. For normal tissues, we use constraints proposed by Kong *et al*, the Radiation Therapy Oncology Group (RTOG) 0236 and 0813 guidelines, and the NRG BR-001 guidelines (provided in Supplementary file 1) [42-44]. If patients received chemotherapy after SRT, data was gathered about the agents used and the number of cycles.

### Follow-up

Patients were seen in the clinic at 1 month after completion of their treatment, then every 3 months for the first year, then every 6 months until May 1, 2015. Imaging, adverse events, and compliance of all patients were monitored during this follow-up period using our clinical databases.

### Endpoints

The primary end-point was local control rate (LC; defined as no progression of treated disease on follow-up scans), which was categorized as complete response (CR), partial response (PR), or stable disease (SD) using the RECIST 1.1 Response Evaluation Criteria in Solid Tumors [45]. Patients were considered to have a local failure if there was evidence of increased size of enhancing tumor in the treated region. PET-CT scan was employed to assist with differentiating radiation-related changes from local or regional recurrence. LC was assessed at a minimum of 6 months after SRT, in order to avoid uncertainty associated with early transient radiographic changes within the high-dose region.

The secondary end-points were: (1) the time to symptom alleviation (defined as the time between the date of SRT completion and the date of symptom alleviation or the date of the last follow-up for censored patients); (2) overall survival (OS, defined as the time between the date of the SRT and the date of death or the date of the last follow-up for censored patients); (3) pattern of failure, including locoreginal failure and/or distant metastases; and (4) Common Terminology Criteria for Adverse Events (CTCAE v4.0) grade toxicity. All toxicities were assessed in a multidisciplinary setting.

### Statistical analysis

LC and OS curves were estimated using Kaplan-Meier analysis and compared using the stratified log-rank test, with *p* value ≤ 0.05 considered statistically significant. Data were analyzed using the statistical software Intercooled Stata version 8.2 for Windows (Stata Corporation, College Station, Texas, USA).

## SUPPLEMENTARY MATERIAL FILES


