# Supplementary material for: Stereotactic radiation therapy for oligometastases or oligorecurrence within mediastinal lymph nodes
Source: Oncotarget. 2016 Feb 23;7(14):18135–45. doi: 10.18632/oncotarget.7636 (PMC4951277; doi:10.18632/oncotarget.7636)
Supplement: Supplementary file 1 [file oncotarget-07-18135-s001.pdf]

Stereotactic radiation therapy for oligometastases or oligorecurrence within mediastinal lymph nodes

Supplementary Material

Supplementary file 1: The normal tissue constraints of the mediastinum for planning SRT

Organs/Tissue at risk	Dose limiting				
	RTOG 0236	RTOG 0813	NRG-BR001		Avoidance endpoint
Spinal cord	Volume: any point<br>Dose (cGy): 18 Gy (6 Gy/fx)	Volume: < 0.25 cc<br>< 0.5 cc<br>Volume max (Gy): 22.5 Gy (4.5 Gy/fx)<br>13.5 Gy (2.7 Gy/fx)<br>Max point dose (Gy): 30 Gy (6 Gy/fx)	3 fx:<br>Volume: < 0.03 cc<br>Volume dose (Gy): 22.5<br>Volume: < 1.2 cc<br>Volume dose (Gy): 13	5 fx:<br>Volume: <0.03 cc<br>Volume dose (Gy): 22.5<br>Volume: <0.35 cc<br>Volume dose (Gy): 22<br>Volume: <1.2 cc<br>Volume dose (Gy): 15.6	Myelitis
Esophagus	Volume: any point<br>Dose (cGy): 27 Gy (9 Gy/fx)	-	3 fx:<br>Volume: < 0.03 cc<br>Volume dose (Gy): 27<br>Volume: < 5 cc<br>Volume dose (Gy): 17.7	5 fx:<br>Volume: < 0.03 cc<br>Volume dose (Gy): 35<br>Volume: < 5 cc<br>Volume dose (Gy): 27.5	Stenosis/fistula
Ipsilateral brachial plexus	Volume: any point<br>Dose (cGy): 24 Gy (8 Gy/fx)	Volume: < 3 cc<br>Volume max (Gy): 30 Gy (6 Gy/fx)<br>Max point dose (Gy): 32 Gy (6.4 Gy/fx)	3 fx:<br>Volume: < 0.03 cc<br>Volume dose (Gy): 26<br>Volume: < 3 cc<br>Volume dose (Gy): 22	5 fx:<br>Volume: < 0.03 cc<br>Volume dose (Gy): 32<br>Volume: < 3 cc<br>Volume dose (Gy): 30	Neuropathy
Heart/Pericardium	Volume: any point<br>Dose (cGy): 30 Gy (10 Gy/fx)	-	3 fx:<br>Volume: < 0.03 cc<br>Volume dose (Gy): 30<br>Volume: < 15 cc<br>Volume dose (Gy): 24	5 fx:<br>Volume: < 0.03 cc<br>Volume dose (Gy): 38<br>Volume: < 15 cc<br>Volume dose (Gy): 32	Neuropathy

Supplementary file 1: (Continued) The normal tissue constraints of the mediastinum for planning SRT

Organs/Tissue at risk	Dose limiting				
	RTOG 0236	RTOG 0813	NRG-BR001		Avoidance endpoint
Trachea and ipsilateral bronchus	Volume: any point<br>Dose (cGy): 30 Gy (10 Gy/fx)	-	3 fx:<br>Volume: < 0.03 cc<br>Volume dose (Gy): 30<br>Volume: < 5 cc<br>Volume dose (Gy): 25.8	5 fx:<br>Volume: < 0.03 cc<br>Volume dose (Gy): 40<br>Volume: < 5 cc<br>Volume dose (Gy): 32	Stenosis/fistula
Whole lung (right & left)	-	Volume: < 1000 cc<br>Volume max (Gy): 13.5 Gy (2.7 Gy/fx)<br>Max point dose (Gy): -	3 fx:<br><15% lung volume (Gy): 20<br><37% lung volume (Gy): 11	5 fx:<br><37% lung volume (Gy): 13.5<br><1500 cc (Gy): 12.5<br><1000 cc (Gy): 13.5	Pneumonitis
Skin	-	Volume: < 10 cc<br>Volume max (Gy): 30 Gy (6 Gy/fx)<br>Max point dose (Gy): 32 Gy (6.4 Gy/fx)	3 fx:<br>Volume: < 0.03 cc<br>Volume dose (Gy): 33<br>Volume: < 10 cc<br>Volume dose (Gy): 31	5 fx:<br>Volume: < 0.3 cc<br>Volume dose (Gy): 38.5<br>Volume: < 10 cc<br>Volume dose (Gy): 36.5	Ulceration

Supplementary file 2: Summary of patient characteristics of NSCLC

Parameter	Group A (17 pts)	Group B (36 pts)	All (53 pts)
Age (years)			
< 60	8 (47.1%)	16 (44.4%)	24 (45.3%)
≥ 60	9 (52.9%)	20 (55.6%)	29 (54.7%)
Gender			
Male	12 (70.6%)	22 (61.1%)	34 (64.2%)
Female	5 (29.4%)	14 (38.9%)	19 (35.8%)
Pathology of primary cancer			
Squamous cell carcinoma	7 (41.2%)	22 (61.1%)	29 (54.7%)
Adenocarcinoma	4 (23.5%)	12 (33.3%)	16 (30.2%)
Large cell carcinoma	2 (11.8%)	2 (5.6%)	4 (7.5%)
Other[†]	4 (23.5%)	0	4 (7.5%)
Location of primary cancer			
Right upper lobe	2 (11.8%)	12 (33.3%)	14 (26.4%)
Right middle lobe	1 (5.9%)	2 (5.6%)	3 (5.7%)
Right lower lobe	3 (17.6%)	3 (8.3%)	6 (11.3%)
Left upper lobe	7 (41.2%)	13 (36.1%)	20 (37.7%)
Left lower lobe	4 (23.5%)	6 (16.7%)	10 (18.9%)
Radiographic appearanceof primary cancer			
Nodular	16 (94.1%)	30 (83.3%)	46 (86.8%)
Infiltrative	1 (5.9%)	6 (16.7%)	7 (13.2%)
Initial stage[‡]			
I/II	1 (5.9%)	27 (75%)	28 (52.8%)
IIIA	5 (29.4%)	9 (25%)	14 (26.4%)
IIIB	11 (64.7%)	0	11 (20.8%)
No. of MLNs within LN zone			
1	16 (94.1%)	31 (86.1%)	47 (88.7%)
2	1 (5.9%)	5 (13.9%)	6 (11.3%)
MLN type			
Recurrence	12 (70.6%)	11 (30.6%)	23 (43.4%)
Second primary	5 (29.4%)	25 (69.4%)	30 (56.6%)

Clinical symptoms of MLNs			
Yes	12 (70.6%)	15 (41.7%)	27 (50.9%)
No	5 (29.4%)	21 (58.3%)	26 (49.1%)
Synchronous metastases			
Yes	3 (17.6%)	26 (72.2%)	21 (39.6%)
No	14 (82.4%)	10 (27.8%)	32 (60.5%)
Radiographic diagnosis MLNs			
PET-CT	10 (58.8%)	28 (77.8%)	38 (71.7%)
CT	7 (41.2%)	8 (22.2%)	15 (28.3%)
Recurrent staging‡			
II	0	3 (8.3%)	3 (5.7%)
III	14 (82.4%)	23 (63.9%)	37 (69.8%)
IV	3 (17.6%)	10 (27.8%)	13 (24.5%)
Interval between the diagnosis of primary and SRT (mths)			
< 12.5	3 (17.6%)	21 (58.3%)	24 (45.3%)
≥ 12.5	14 (82.4%)	15 (41.7%)	29 (54.7%)
Therapy prior to SRT§			
S	17 (100%)	23 (63.9%)	40 (75.5%)
CT	14 (82.4%)	18 (50%)	32 (60.4%)
RT	0	36 (100%)	36 (67.9%)
MTT	0	3 (8.3%)	3 (5.7%)
SRT treatment intent			
Curative	17 (100%)	35 (97.2%)	52 (98.1%)
Palliative	0	1 (2.8%)	1 (1.9%)
Therapy after SRT§			
CT	8 (47.1%)	21 (58.3%)	29 (54.7%)
MTT	3 (17.6%)	1 (2.8%)	4 (7.5%)
None	6 (35.3%)	14 (38.9%)	20 (37.8%)

Note: Group A: Patients with MLNs who received treatment including surgery and/or chemotherapy and/or molecular targeted therapy, but not radiation therapy before SRT; Group B: Patients with MLNs who received treatment including surgery and/or chemotherapy and/or molecular targetd therapy, and radiation therapy.

† Other is defined as the various combinations of squamous cell carcinoma, adenocarcinoma, and large cell carcinoma.

‡The AJCC (6th edition) was used for staging.

§ Some patients had more than one therapy.

Abbreviations: Pts: patients; MLNs: mediastinal lymph node metastases; PET-CT: positron emission tomography/computed tomography; CT: computed tomography; SRT: stereotactic radiation therapy; S: surgery; CT: chemotherapy; RT: radiotherapy; MTT: molecular targeted therapy; mths: months.

Supplementary file 3: The radiation dose for esophagus, trachea, and heart with median for MLN.

		Tachea (Gy)						Esophagus (Gy)						Heart (Gy)					
Nodal zone	No. (%)[†]	D_{max}	$D_{0.1}$	$D_{0.2}$	D_1	D_2	D_5	D_{max}	$D_{0.1}$	$D_{0.2}$	D_1	D_2	D_5	D_{max}	D_5	D_{10}	D_{20}	D_{30}	D_{40}
Upper	46 (47%)																		
1R	5 (11%)	10.2	7.5	6.9	6.9	6.4	5.8	11.9	11.5	11.0	10.4	10.4	9.2	2.3	1.2	0.7	0.6	0	0
1L	4 (9%)	15.5	10.4	9.8	8.6	8.2	7.4	16.7	16.2	15.9	15.6	15.2	14.7	1.5	1.4	1.0	0.7	0	0
2R	14 (30%)	42.1	36.8	29.8	25.1	19.9	12.3	44.7	33.3	21.1	15.8	11.7	7.6	9.9	2.3	1.2	0.7	0.6	0
2L	3 (7%)	47.2	41.1	34.2	30.7	24.4	17.5	41.2	30.5	19.7	13.1	9.2	8.9	12.1	6.4	2.1	1.5	1.0	0.7
3A	6 (13%)	8.9	7.7	6.7	6.2	5.6	3.6	6.9	6.7	6.2	5.6	5.1	3.6	48.4	30.8	23.1	15.9	12.8	10.8
4R	8 (17%)	40.5	35.1	32.2	30.8	28.4	24.6	21.1	14.7	12.3	11.4	9.9	7.6	37.7	33.6	29.8	27.9	25.6	21.3
4L	6 (13%)	43.8	40.9	37.6	35.5	31.1	22.4	20.3	16.9	13.6	12.6	10.9	8.2	43.6	40.9	37.6	34.9	30.0	21.3
Aorticopulmonary	21 (21%)																		
5	17 (81%)	55.8	50.4	47.4	45.0	40.3	29.2	23.5	20.5	17.4	16.6	15.8	14.2	58.2	45.8	41.1	34.0	28.4	25.3
6	4 (19%)	15.3	13.7	11.4	9.7	8.0	5.7	19.7	16.5	13.1	11.4	9.7	7.4	54.5	46.1	42.7	36.5	32.5	25.6
Subcarinal	9 (9%)																		
7	9 (100%)	46.3	42.2	36.1	32.0	25.9	14.3	51.7	47.6	42.2	38.1	31.3	16.3	51.2	34.0	27.9	21.1	17.7	15.7
Lower	7 (7%)																		
8	2 (29%)	16.6	15.1	13.9	13.4	12.3	12.3	16.7	15.1	13.9	12.9	11.2	8.4	4.2	0	0	0	0	0
9	5 (71%)	17.4	16.5	14.9	14.5	13.4	13.0	8.2	7.6	6.6	6.0	5.5	4.4	3.9	0	0	0	0	0
Hilar-interlobar	15 (15%)																		
10R	8 (53%)	23.5	19.9	16.1	14.6	13.6	10.7	28.7	26.8	24.3	22.9	19.9	16.5	45.5	38.0	36.5	33.6	31.1	28.7
10L	7 (47%)	21.4	18.6	15.2	12.9	10.2	9.7	28.1	23.4	19.3	16.4	13.4	9.4	50.9	33.9	16.3	19.3	17.0	15.8
All	98 (100%)																		

[†] Number of MLN metastases. Abbreviations: MLNs: mediastinal lymph nodes; R: right; L: left.
